# Role of Tissue Factor in *Mycobacterium tuberculosis-*Induced Inflammation and Disease Pathogenesis

**DOI:** 10.1371/journal.pone.0114141

**Published:** 2014-12-02

**Authors:** Hema Kothari, Shiva Keshava, Rit Vatsyayan, Nigel Mackman, L. Vijaya Mohan Rao, Usha R. Pendurthi

**Affiliations:** 1 Department of Cellular and Molecular Biology, The University of Texas Health Science Center at Tyler, Tyler, TX 75708, United States of America; 2 Division of Hematology and Oncology, McAllister Heart Institute, Department of Medicine, University of North Carolina at Chapel Hill, Chapel Hill NC 27599, United States of America; The Catholic University of the Sacred Heart, Rome, Italy

## Abstract

Tuberculosis (TB) is a chronic lung infectious disease characterized by severe inflammation and lung granulomatous lesion formation. Clinical manifestations of TB include hypercoagulable states and thrombotic complications. We previously showed that *Mycobacterium tuberculosis* (*M.tb*) infection induces tissue factor (TF) expression in macrophages *in vitro*. TF plays a key role in coagulation and inflammation. In the present study, we investigated the role of TF in *M.tb*-induced inflammatory responses, mycobacterial growth in the lung and dissemination to other organs. Wild-type C57BL/6 and transgenic mice expressing human TF, either very low levels (low TF) or near to the level of wild-type (HTF), in place of murine TF were infected with *M.tb via* aerosol exposure. Levels of TF expression, proinflammatory cytokines and thrombin-antithrombin complexes were measured post *M.tb* infection and mycobacterial burden in the tissue homogenates were evaluated. Our results showed that *M.tb* infection did not increase the overall TF expression in lungs. However, macrophages in the granulomatous lung lesions in all *M.tb*-infected mice, including low TF mice, showed increased levels of TF expression. Conspicuous fibrin deposition in the granuloma was detected in wild-type and HTF mice but not in low TF mice. *M.tb* infection significantly increased expression levels of cytokines IFN-γ, TNF-α, IL-6 and IL-1ß in lung tissues. However, no significant differences were found in proinflammatory cytokines among the three experimental groups. Mycobacterial burden in lungs and dissemination into spleen and liver were essentially similar in all three genotypes. Our data indicate, in contrast to that observed in acute bacterial infections, that TF-mediated coagulation and/or signaling does not appear to contribute to the host-defense in experimental tuberculosis.

## Introduction

Tuberculosis (TB) is a chronic lung infectious disease caused by *Mycobacterium tuberculosis* (*M.tb*). Although there has been a decline in the emergence of new TB cases and TB-associated mortality, TB still remains one of the world’s most prevalent infectious diseases [1]. Among other clinical presentations, increasing evidence indicates the presence of hematologic abnormalities, particularly disseminated intravascular coagulation (DIC) and deep-vein thrombosis (DVT), in TB patients [2]–[4]. The hemostatic and inflammatory changes in TB can result in a hypercoagulable state [3], [5]. The prevalence of venous thromboembolism ranges from 0.6% to 3% in TB patients [6] and clinical reports emphasize that patients with severe pulmonary TB are at risk of developing thromboembolic events [7].

Tissue factor (TF) is a transmembrane receptor that binds plasma factor VII/VIIa and triggers the blood coagulation after vascular injury and in various diseases [8], [9]. TF is not normally expressed in cells that come in direct contact with blood [10], [11]. However, various bacterial and viral infections can trigger TF expression on monocytes and endothelial cells [12], [13]. Elevated TF coagulant activity on vascular cells and circulating TF-positive microparticles lead to enhanced thrombosis [9], [14], [15]. Additionally, TF beyond its procoagulant function also facilitates protease activated receptors (PAR)-mediated cell signaling, either directly or by downstream generation of thrombin [16], [17]. Aberrant TF expression and the resultant coagulation activation is a major cause for infection-associated mortality and inflammation [18]. Inhibition of the TF-dependent coagulation has been shown to reduce inflammation and improve survival in animal models of bacterial sepsis and various virus infection models [12], [19]. Interestingly, in contrast to its damaging effects, TF-mediated extrinsic coagulation pathway can also play a protective role in host-defense against certain bacterial infections by reducing pathogen burden and limiting their capacity to disseminate [20]–[23]. Earlier studies reported that mycobacterial cell wall components can induce TF expression in macrophages [24]–[27]. More recently, we showed that both gamma-irradiated (γ-*Mtb*) and live *M.tb* (H37Rv) infection markedly upregulates TF expression and procoagulant activity in macrophages and endothelial cells [28]. At present, it is unknown whether TF expression has any functional role in TB pathogenesis.

One of the hallmark features of TB pathology is the development of granuloma which signifies immune-mediated containment of the infection [29]. Granulomas are organized immune aggregates consisting of blood-derived *M.tb-*infected and uninfected macrophages, foamy macrophages, epithelioid cells, giant-multinucleated cells, lymphocytes and fibroblasts [30]. In a later stage, granuloma formation is accompanied by fibrosis, which restricts the escape of pathogen and inflammatory mediators from the localized areas in the lung, and therefore confines the infection [31], [32]. A recent study, where mycobacterial trehalose dimycolate was administered to wild-type and fibrinogen knockout mice subcutaneously, indicated that fibrinogen promotes granulation tissue formation [33]. Since TF is the key element in generation of fibrin, it is possible that *M.tb*-induced TF expression on the surface of activated macrophages may play a role in the containment of mycobacterial infection through generation of fibrin. It is also possible that TF could influence *M.tb* pathogenesis through its signaling function. Therefore, in the present study, we sought to determine the role of TF in *M.tb*-induced inflammatory responses, mycobacterial growth and containment of infection using transgenic mice that express either very low levels of human TF or high levels of human TF in place of murine TF.

## Materials and Methods

### Ethics statement

Animals: All studies involving animals were conducted in accordance with the animal welfare guidelines set forth in the Guide for the Care and Use of Laboratory Animals and Department of Health and Human Services, and approved by the Institutional Animal Use and Care Committee of the University of Texas Health Science Center at Tyler, Tyler TX (Animal Welfare Assurance Number A3589-01; Protocol Number: 491).

### Animals

C57BL/6 wild-type mice were obtained from Jackson Laboratory (Bar Harbor, ME) or internal breeding program. The generation of low TF and human TF (HTF) mice was described earlier [34], [35]. 6–8 weeks old male and female mice were used for all studies. Animal studies were approved by the Institutional Animal Use and Care, and Infectious Organism Research Review committees of the institute. Humane end points were used in the study. Mice were observed on a daily basis throughout the experimental period by a well-trained vivarium staff. Visible signs for pain or distress were used as human endpoints. Rapid respiration, noticeable weight loss, dehydration (visible vertebrae in the tail region), hunch-back appearance, and immobility were used as signs of pain and distress. None of the mice showed these symptoms.

### Reagents

Gamma-irradiated H37Rv (γ-*Mtb*) and *Mycobacterium tuberculosis* (*M.tb*) H37Rv (27294) were obtained from ATCC (Rockville, MD). Recombinant human factor VIIa (FVIIa) was from Novo Nordisk (Gentofte, Denmark). Human factor X was purchased from Enzyme Research Laboratories (South Bend, IN). Chromogenic substrate Chromogenix S-2765 was from DiaPharma (West Chester, OH). Cytokine ELISA kits were from eBioscience (San Diego, CA). Mouse TAT-complex ELISA kit was from Assaypro (St. Charles, MO). Mouse macrophage marker antibody F4/80 (SP115) was from Novus Biologicals (Littleton, CO) and neutrophil marker Ly-6G (IA8) was from BD Pharmingen (San Diego, CA). Preparation and characterization of monospecific polyclonal antibodies against human TF were described earlier [36]. Rabbit polyclonal anti-mouse TF antibody was kindly provided by Lars C. Petersen, Novo Nordisk, Denmark.

### Mycobacterial culture and aerosol infection in mice

*M.tb* culture and stock storage conditions were same as described earlier [28]. For experimental infections, frozen aliquots of *M.tb* H37Rv was thawed, washed in phosphate-buffered saline (PBS) and diluted in 10 ml of sterile PBS. Mice were infected with *M.tb* H37Rv in an aerosol exposure chamber as described earlier [37]. *M.tb* dose given to mice was selected by exposing mice to varying concentrations of *M.tb* and analyzing CFU counts in homogenized lungs 24 h post infection. *M.tb* concentration of 2×10^7^ CFU/ml led to deposition of ∼50–100 bacteria in the lungs per mouse. This dose was selected for further mice infections. *M.tb* infections were performed three independent times and 3–7 animals per group were used at each time.

### Bronchoalveolar lavage and macrophage isolation

Mice were euthanized and lungs were flushed with 1 ml of sterile PBS containing 0.5 mM EDTA following cannulation of their trachea. The 1 ml BAL fluid was aspirated and centrifuged at 5000 g for 5 min to remove cells. The supernatants were frozen at −80°C until used for cytokine measurements. For isolation of alveolar macrophages, lungs were flushed with an additional 5 ml of PBS/EDTA as described above. Cells were pelleted by centrifugation at 5000 g for 10 min and treated with the red blood cell (RBC) lysis solution to remove an occasional RBC contamination. Cells were washed, resuspended in RPMI complete medium, and plated in 96-well culture plate.

### Measurement of bacterial burden

Mice were sacrificed at 2 and 8 weeks post infection. Lung, liver and spleen were aseptically removed and homogenized manually in sterile saline (lung and spleen in 500 µl; liver in 2 ml). Organ homogenates (10× and 100× dilutions of spleen and liver; 500× and 2000× dilutions of lungs) were then plated onto 7H11 agar plates to determine bacterial burden. CFUs were counted after 21 days of incubation at 37°C. CFUs are represented per organ. A portion of lung from 3–5 mice in each experimental group was reserved for histology.

### Lung histopathology and immunohistochemistry

Before excising lungs, number of visual lung lesions in all lobes was counted in *M.tb-*infected mice. Lungs were fixed in Excell plus fixative (American MasterTech Scientific Inc.) for at least 48 h before processing them for paraffin embedding. Thin sections (5 µm) were stained with hematoxylin and eosin (H&E) for histopathological examination. Immunohistochemistry was performed essentially as described in an earlier publication [38]. H&E and immune-stained sections were viewed under Olympus BX50 microscope and photographed with Olympus UCMAD3 camera using Picture Frame software. The same exposure settings were used for all image capturing. For determination of granuloma size, images captured at 4× magnification were used to measure granuloma diameter using Nikon NIS-Elements BR 3.2 software. TF expression levels in the granuloma was estimated based on the intensity of the immunopositive areas by scoring semi-quantitatively as absent, slight, moderate or strong with assigned scores of 0, 1, 2 and 3, respectively. Total 10–20 granulomas were analyzed to calculate either average granuloma size or scoring TF staining intensity by two investigators.

### Measurement of cytokines and TAT levels

Cytokines in lung homogenates and BAL, and TAT levels in plasma and BAL were measured by ELISA according to manufacturer’s instructions.

### TF activity analysis in lung homogenates

Lung homogenates were diluted 1∶1 in buffer A (10 mM Hepes, 0.15 M NaCl, 4 mM KCl, 11 mM glucose, pH 7.5) containing 20 mM octyl β-D-glucopyranoside. TF activity in lung homogenates was measured by adding either mouse FVIIa (for wild-type) or human FVIIa (for low TF and HTF mice) (10 nM) and human FX (175 nM) and measuring FXa generation in a chromogenic assay as described earlier [39].

### Isolation, culture and *ex vivo M.tb* infection of mouse macrophages

Bone-marrow-derived macrophages and resident peritoneal macrophages were prepared from 6–8 week old wild-type, HTF and low TF mice as described [40]. Peripheral blood mononuclear cells (PBMCs) from whole blood were isolated by density gradient using Ficoll-paque PLUS. Cells were cultured in serum-rich RPMI medium containing 10 ng/ml recombinant mouse macrophage colony stimulating factor (MCSF) for 4 days at 37°C. Before infection, macrophages were washed once with Hanks-buffered salt solution and fresh complete medium without any antibiotics was added to the cells. Macrophages were then challenged with either live H37Rv (10 CFU/cell) or *γ-Mtb* (10 µg/ml) for overnight. Next day, cell surface macrophage TF activity was analyzed as described earlier [28]. Same number of macrophages was cultured for the square culture area for TF activity analyses.

### Statistics

The data were shown as the mean ± SEM. Statistical significance between the two experimental groups was determined by Students t-test. One-way analysis of variance was used to determine statistical significance among three groups.

## Results

### Analysis of TF expression in lungs of wild-type, HTF and low TF mice

**Figure 1:**
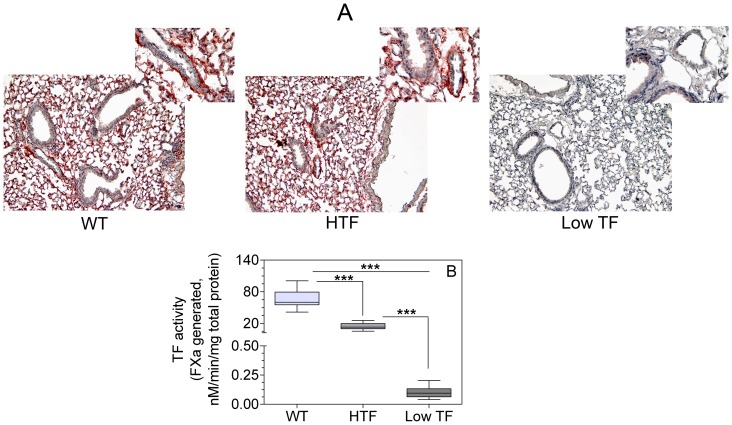
Tissue factor expression levels in wild-type (WT), HTF and low TF mice. (**A**) Immunohistochemical analysis of TF expression in WT, HTF, and low TF mice. Representative images (magnification, 10×; inset, 40×) are shown. TF immunohistochemical staining was performed using anti-murine TF antibodies for WT mice and anti-human TF antibodies for HTF and low TF mice. (**B**) TF activity of total lung homogenates of WT, HTF, and low TF mice. TF activity was measured in FX activation assay using species-specific FVIIa. (n = 9–14 per group). *** denotes p<0.0001 as determined by one-way ANOVA and student t-test.

First, we performed a parallel comparative analysis of the TF expression and procoagulant activity in lungs of wild-type, HTF and low TF mice. Immunohistochemical analysis of TF protein expression (Fig. 1A) showed intense staining for TF antigen in the lungs of wild-type and HTF mice. In comparison, TF staining in lungs of low TF mice was negligible. TF activity in the lung extracts of low TF mice was>100 times lower compared to that of HTF and wild-type mice (Fig. 1B), which is consistent with earlier reported data [34]. Levels of TF activity in the lung extracts of HTF mice were ∼25% of those observed in wild-type mice. This is similar to the reported 40% level observed in an earlier study [35].

### Role of TF in tuberculosis pathogenesis

#### (i) Lung lesions

**Figure 2:**
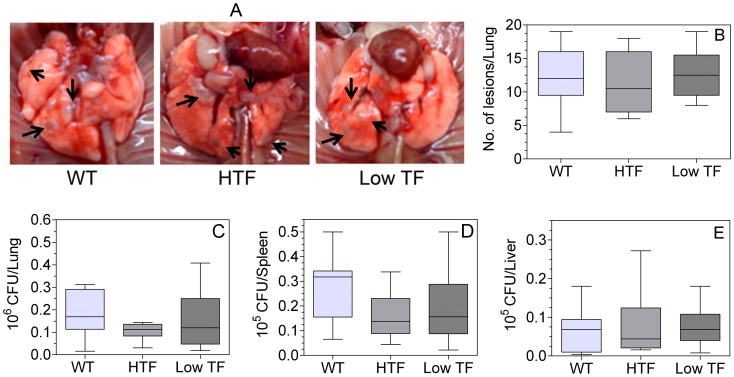
TF deficiency does not affect lung lesions, mycobacterial growth and dissemination in mice infected with *M.tb.* (**A**) Representative images of whole lungs after 8 weeks of *M.tb* aerosol exposure showing grey-white nodule like lesions (arrow marks). (**B**) Visible lesions on the lung surface of each lobe were counted and shown as an average plot. Mycobacterial burden in lungs (**C**) and dissemination into spleen (**D**) and liver (**E**) was determined as described in methods. Data are representative of three independent experiments (total n = 9–19 per group). No significant differences were found among the groups.

Gross examination of the lungs showed no visual lung lesions after 2 weeks of *M.tb* infection in all three genotypes (data not shown). However, at eight-week post infection, considerable inflammation in the lungs was evident by the presence of distinct macroscopic grey-white lung lesions on the surface of lungs (Fig. 2A). No difference in the number of macroscopic lung lesions, which varied from 6–19 lesions per lung, was observed among *M.tb-*infected wild-type, HTF and low TF mice (Fig. 2B).

#### (ii) Mycobacterial growth and dissemination

Enumeration of the lung bacterial loads showed no significant differences between wild-type, HTF and low TF mice at 2 weeks (data not shown) and 8 weeks (Fig. 2C). There was a wide variation in lung bacterial burden among individual animals within a group, and the CFUs varied from 0.05–0.4×10^6^/lung. Mycobacterial dissemination to extrapulmonary sites was determined by quantitation of bacterial CFUs in the spleen and liver. Experiments performed with a limited number of animals at 2 weeks showed very few CFUs (average <200) in the spleen and liver of all three genotypes, and no CFUs were detected in ∼50% of the mice in all experimental groups. Although, there was a marked increase in liver and splenic bacterial burden from 2 to 8 weeks, TF deficiency appears to have no impact on mycobacterial dissemination as low TF mice displayed similar CFU counts in the spleen and liver as wild-type and HTF mice (Fig. 2D and 2E).

#### (iii) Granuloma

**Figure 3:**
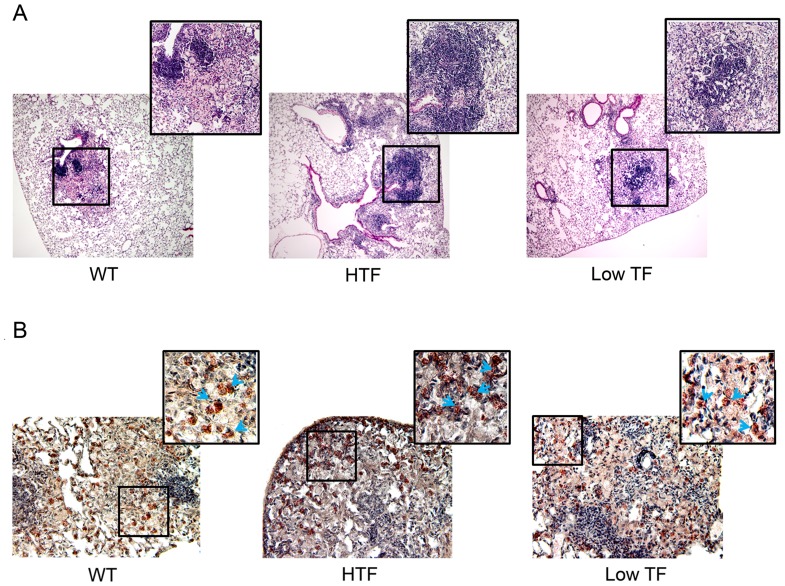
TF deficiency has limited impact on *M.tb*-induced lung inflammation and histopathology. Eight-week post infection, lungs were removed, fixed in Excell plus fixative, sectioned, and stained with H&E or immunostained for macrophages. (**A**) Representative H&E images of wild-type (WT), HTF and low TF mice lungs showing granulomatous lesions (magnification, 4×; inset, 10×). (**B**) Granuloma tissue sections were immunostained with macrophage marker F4/80 antibody (magnification, 20×; inset, digitally zoomed). Images are representative of 3–5 mice per group. Blue arrow heads point macrophages stained (red color) with F4/80 antibody.

Histologic examination by H&E staining showed marked infiltration of inflammatory cells into the alveolar spaces and formation of dense granulomatous lesions in the lungs of *M.tb-*infected mice (Fig. 3A). Although the average size of the granulomas appeared to be slightly smaller in low TF mice compared to wild-type and HTF mice (wild-type, 734±49.7; HTF, 715±120.8; and low TF, 660±63.9 µm diameter), the differences among them were not statistically significant. Qualitatively, granulomas in the wild-type and HTF mice appeared to be more organized whereas the granuloma structure in the low TF mice was comparatively sparse and disorganized. Immunohistochemical staining for F4/80 displayed similar distribution of macrophages dispersed throughout the normal lung tissue as well as inside the granulomas in all genotypes (Fig. 3B). Similarly, no differences were observed in neutrophil infiltration into lungs among the genotypes at 8 weeks post infection (data not shown).

#### (iv) Cytokine response

**Figure 4:**
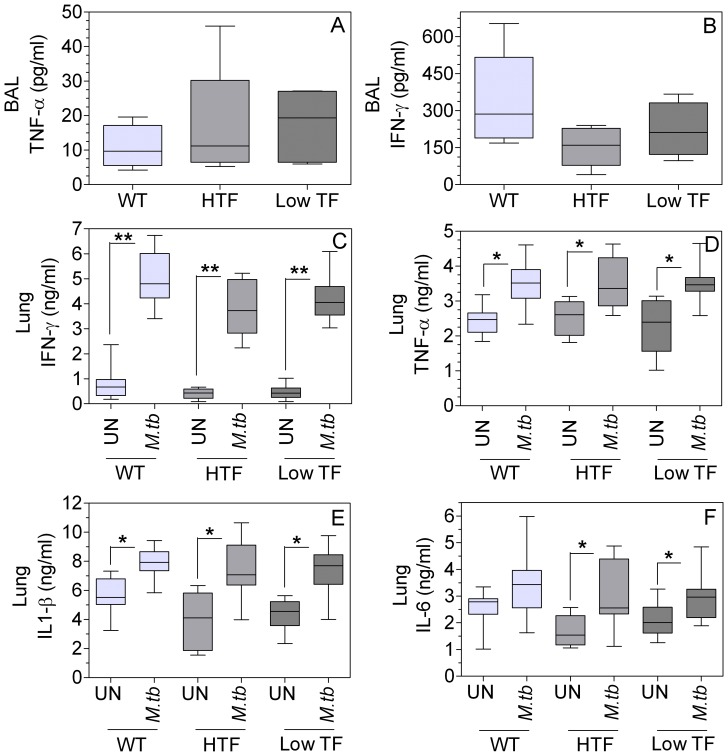
TF deficiency does not affect the proinflammatory cytokine response to *M.tb* infection. Cytokine levels in BAL fluids (**A, B**) and lung homogenates (**C–F**) were measured in ELISA. (**A**) TNF-α levels in BAL; (**B**) IFN-γ levels in BAL; (**C**) IFN-γ levels in lung homogenates; (**D**) TNF-α levels in lung homogenates; (**E**) IL-1-ß levels in lung homogenates; (**F**) IL-6 levels in lung homogenates. UN, uninfected; *M.tb*, infected with live H37Rv. (n = 8–19 per group). * denotes significantly different from uninfected controls. (p<0.05) as obtained by one-way ANOVA and Tukey post-test.

Cytokine analysis of BAL fluids 8 weeks after *M.tb* infection showed increased levels of TNF-α and IFN-γ in all genotypes compared to their uninfected controls (levels in the uninfected controls were below the ELISA detection limit), but no significant differences among genotypes were observed (Fig. 4A and 4B). IL1-β and IL-6 levels in BAL fluids of both uninfected and *M.tb-*infected mice were below the ELISA detection limits of 8 pg/ml and 4 pg/ml, respectively. The proinflammatory cytokine profile of lung homogenates of *M.tb-*infected wild-type, HTF and low TF mice showed significantly increased levels of all four cytokines as compared to uninfected controls in all genotypes (Fig. 4C–F). IFN-γ levels increased 9-fold (Fig. 4C) and TNF-α, IL1-β and IL-6 levels were increased about 2-fold (Fig. 4D–F) in lungs following *M.tb* infection. Similar to BAL fluids, no significant differences were observed in all four cytokines in lung homogenates of wild-type, HTF and low TF mice at 8 weeks post *M.tb* infection.

#### (v) TF expression and procoagulant activity

**Figure 5:**
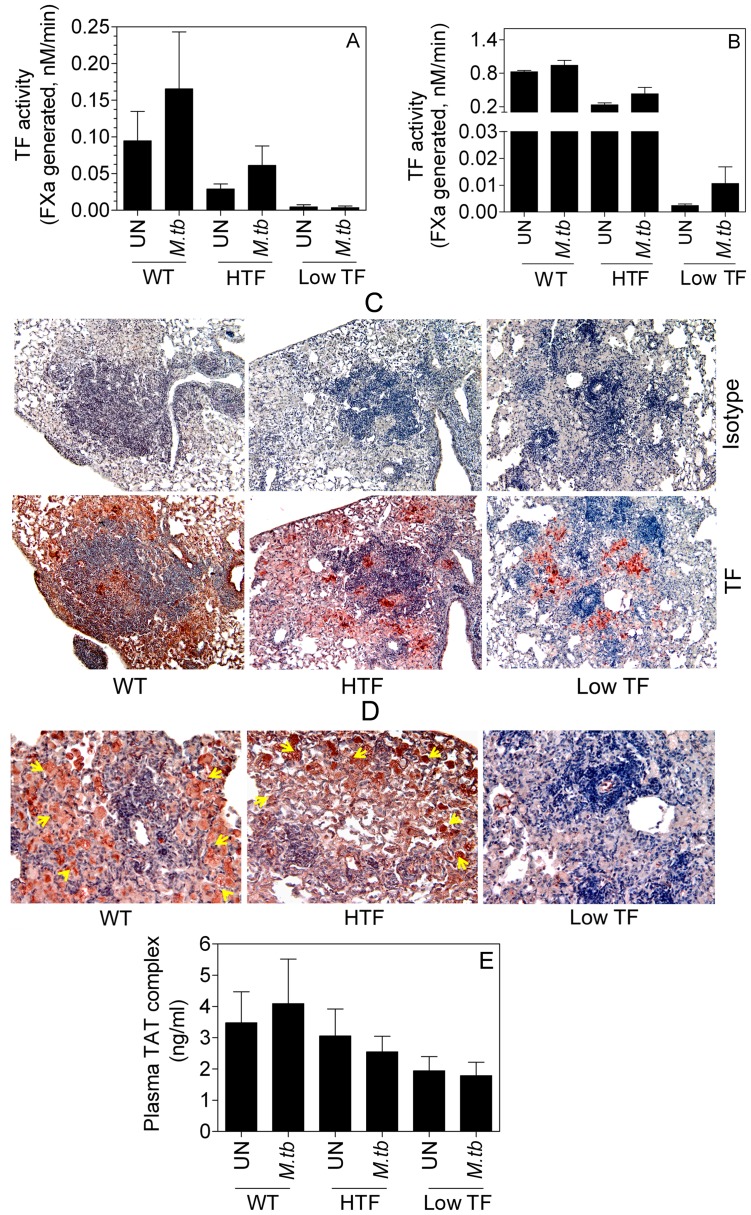
TF expression in alveolar macrophages, lungs, and granuloma upon *M.tb* infection. Eight weeks post infection, (**A**) alveolar macrophages were harvested from BAL and TF procoagulant activity was measured (**B**) TF activity of the lung homogenates of uninfected and *M.tb-*infected mice. Data are mean ± SEM (n = 5–14 per group). Immunohistochemical analysis of TF expression (**C**) and fibrin generation (**D**) in the granulomas. (**E**) TAT levels in the plasma. Yellow arrow heads point fibrin staining within the granuloma.

Analysis of TF activity in alveolar macrophages isolated after 8 weeks of *M.tb* infection from wild-type and HTF mice showed slightly higher TF procoagulant activity as compared to their uninfected controls, but the increase was not statistically significant (Fig. 5A). Alveolar macrophages from low TF mice had little TF activity compared to the wild-type and HTF mice, and *M.tb.* infection did not increase the activity. We also measured TF activity in the total lung homogenates of *M.tb-*infected and control mice of all genotypes. As shown in Fig. 5B, although not statistically significant, there was a ∼5-fold increase in TF activity in low TF mice lungs upon *M.tb* infection. Infected HTF lung homogenates also showed a modest, but statistically insignificant increase of 1.8-fold.

In additional experiments, we analyzed TF protein expression in lung tissue sections of *M.tb-*infected wild-type, HTF and low TF mice by immunohistochemistry. Analysis of TF expression (Fig. 5C) revealed no apparent difference in normal lung tissues upon *M.tb* infection. Granuloma of wild-type and HTF mice stained intensively positive for TF. Overall, TF expression in the granulomas of low TF mice was significantly lower as compared to that of wild-type and HTF mice. However, intense TF staining was observed in localized areas within the granulomas of low TF mice. The average TF intensity scores of the granulomas calculated as described in methods were as follows- wild-type- 2.214±0.155, HTF- 2.429±0.202 and low TF- 0.955±0.192. The cells stained intensely positive for TF in granuloma of low TF mice and other mice appear to be a subset of macrophages or foam cells, but not lymphocytes. Fibrin staining of the lung sections of *M.tb-*infected mice showed discrete regions/cells in the granulomas of wild-type and HTF mice stained intensely positive (Fig. 5D). Fibrin staining in the granuloma of low TF mice was less intense compared to that of wild-type and HTF mice granulomas.

#### (vi) TAT complex

We determined whether upregulated TF expression in *M.tb* infection is accompanied by changes in systemic and pulmonary coagulation activation by measuring thrombin-antithrombin complexes (TAT). As shown in Fig. 5E, TAT levels in plasma of low TF mice were slightly lower than wild-type and HTF mice. There was no change in the plasma TAT levels upon *M.tb*-infection in all three genotypes. TAT levels in the BAL fluids were also not elevated after *M.tb* infection in wild-type and HTF mice. In contrast, there was a significant 10-fold increase in BAL TAT after *M.tb* infection in low TF mice. Despite this, TAT levels in BAL fluids of low TF mice were markedly lower compared to wild-type and HTF mice (The BAL TAT levels in ng/ml in *M.tb-*infected mice were as follows: wild-type- 147±23.4; HTF- 54±6.6 and low TF- 3.16±0.76).

### Analysis of TF procoagulant activity upon *ex vivo* infection of macrophages with *M.tb*

**Figure 6:**
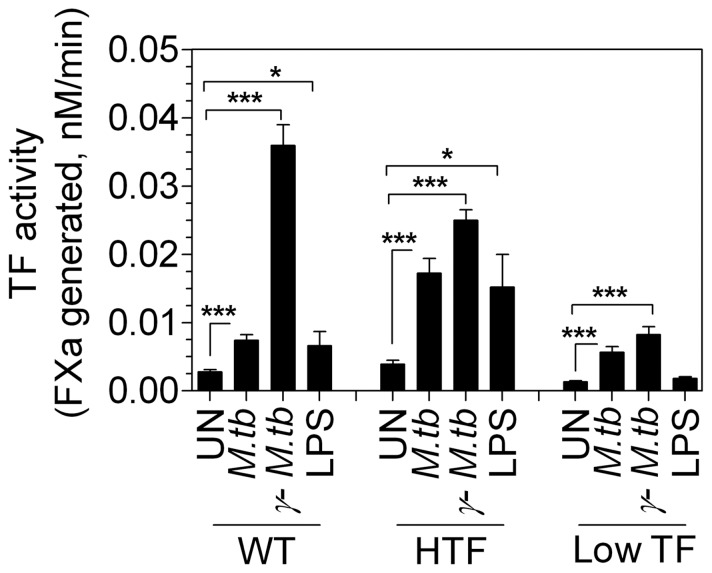
*Ex-vivo M.tb* stimulation increases procoagulant activity of macrophages of wild-type (WT), HTF and low TF. Bone-marrow-derived macrophages (∼0.5×10^5^ cells) were either stimulated with live *M.tb* (10 CFU/cell) or 10 µg/ml of gamma-irradiated *M.tb* (*γ-M.tb*) for overnight in antibiotic free RPMI complete medium. Macrophages were also stimulated with LPS (1 µg/ml) as a positive control for 6 h. At the end of the treatment, TF activity was measured in FX activation assay. * denotes significantly different from unstimulated controls. (* p<0.05; ** p≤0.001; *** p<0.0001) as obtained by Students t-test. Data are mean ± SEM (n = 6–17).

**Figure 7:**
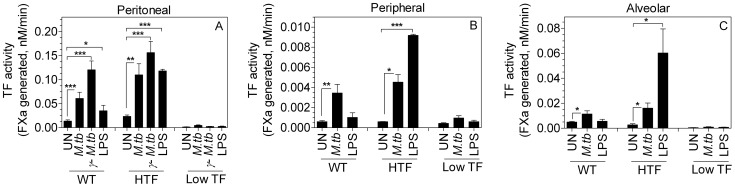
Varied TF expression levels in macrophages derived from different sources in wild-type (WT), HTF and low TF mice in response to *M.tb* and LPS stimulation. Macrophages (∼0.5×10^5^ cells) were either stimulated with live *M.tb* (10 CFU/cell) or 10 µg/ml of gamma-irradiated *M.tb* (*γ-M.tb*) for overnight in antibiotic free RPMI complete medium. Macrophages were also stimulated with LPS (1 µg/ml) as a positive control for 6 h. At the end of the treatment, TF activity was measured in FX activation assay. * denotes significantly different from unstimulated controls. (* p<0.05; ** p≤0.001; *** p<0.0001) as obtained by Students t-test. Data are mean ± SEM (n = 6–17).

Low TF mice express only 1% of TF compared to wild-type and HTF mice but immunohistochemical analysis unexpectedly showed substantial TF expression in the low TF mice granulomas upon *M.tb* infection. Based on immunohistochemical analysis, H&E staining and our earlier results, we believe that the TF expressing cells inside the granulomas are macrophages that are infiltrated into the lung in response to *M.tb.* infection. Therefore, we next investigated whether TF expression can be induced in macrophages of low TF mice by *M.tb* infection. To determine this, we treated bone-marrow-derived macrophages from wild-type, HTF and low TF mice with live *M.tb* and *γ-M.tb ex vivo.* Macrophages were also stimulated with LPS as a positive control. LPS stimulation induced ∼5-fold increase in TF activity in macrophages derived from HTF mice whereas macrophages from low TF mice were nearly unresponsive to LPS (Fig. 6). Interestingly, *γ-M.tb* stimulation led to a 4 to 6-fold increase in TF activity in macrophages derived from both HTF and low TF mice. The difference in TF activity in *M.tb-*infected macrophages of HTF and low TF mice was about 3-fold, which markedly contrasts to a ∼100-fold difference observed in total lung extracts. Additional studies revealed that TF activity in macrophages derived from different sources (peritoneum, peripheral and alveolar) varied considerably and difference in TF activity between *M.tb*-infected macrophages derived from HTF mice and low TF mice varied from 5 to more than 20 fold (Fig. 7).

## Discussion

It is believed that the development of efficient fibrotic response by the host in developing the collagen and fibrin ring-like structure around the granuloma contains the bacteria within the granuloma and limits their capacity to disseminate [41], [42]. Our recent observation that *M.tb* infection markedly upregulates TF expression and increases the procoagulant activity of macrophages [28] raise the possibility that *M.tb*-induced TF expression may play a protective role in TB by promoting fibrin deposition in the granuloma. However, the present study carried out with wild-type, HTF and low TF mice showed that despite subtle differences in the granuloma structure among these mice, no significant differences in *M.tb* growth in the lungs and its dissemination into liver, and spleen was observed among these mice.

*M.tb* infection did not significantly alter the overall TF expression and the activity in the lungs of wild-type and HTF mice. However, *M.tb* infection markedly increased TF expression in localized areas within the granulomas of wild-type and HTF mice. Interestingly, these intensely stained TF-positive areas were also present in the granulomas of low TF mice infected with *M.tb*. This localized increased expression of TF in the granulomas is probably responsible for the substantially increased TF activity in the lung homogenates of low TF mice. Despite this increase, the overall TF expression in lungs of low TF mice, both uninfected and *M.tb*-infected mice, was negligible in comparison to TF levels measured in wild-type and HTF mice.

TF deficiency did not significantly influence *M.tb*-induced proinflammatory cytokine elaboration. These data are in contrast to the data observed in other models of bacteria-induced lung inflammation where blockade of TF function decreases expression of proinflammatory cytokines [43], [44]. It is possible that the close inter-relationship between the coagulation and inflammation may not exist in chronic infections such as TB. Although we cannot rule out the possibility that TF deficiency may have an effect on elaboration of proinflammatory cytokines at the very early stage of the *M.tb* infection, it is important to note here that expression of inflammatory cytokines in mice and guinea pig following *M.tb* infection was shown to occur around 2 to 3 weeks post infection, and the expression was persistent for 10 weeks or more [45], [46].

In line with the published data on *M.tb* pathogenesis in the mouse model [47], we did not observe a distinct fibrotic response encapsulating the granuloma in mice infected with *M.tb.* Although no significant differences were found in the number of the granulomas formed in the lungs of low TF, HTF and wild-type mice infected with *M.tb*, the architecture of the granulomas among these mice appears to be somewhat different. The size of the granulomas appeared to be slightly smaller and mostly disorganized in low TF mice compared to granulomas in HTF and wild-type mice. The increased expression of TF in the granuloma region of wild-type and HTF was associated with fibrin deposition in the granuloma. Small discrete regions of fibrin islands were found peripheral to the core and extending toward the outer margin of the granuloma. This indicates the vascular damage in the granulomatous region. Reduced fibrin was found in granulomas of low TF mice. This probably reflects impaired TF-induced coagulation in these mice rather than lack of vascular damage in the granuloma. Overall, the observation of similar *M.tb* burden and dissemination in low TF mice as compared to wild-type and HTF mice despite marked impairment in fibrin generation in low TF mice suggests that fibrin-mediated physical entrapment, adhesion or immunity may not play a significant role in the pathogenesis of *M.tb,* at least in the mouse model.

T-cells are known to play a crucial role in the protective immune response to TB infection and granulomatous lesion formation [47]. The mechanisms underlying granuloma formation and the containment are highly complex, and involve different T- cell population [48]–[50]. Although we have not investigated here whether low TF and HTF mice infected with *M.tb* have different T-cell response, it is interesting to note that TF-dependent protease-activated receptor-2-mediated signaling is shown to influence T-cell proliferation and cytokine production [51]. Thus, it is possible that the different levels of TF antigen in low TF, HTF and wild-type mice may contribute to differential T-cell response upon *M.tb* infection and this may be the reason for the differences in the granuloma structure that we observed between the genotypes.

A number of studies indicated an association of DVT and DIC in TB patients [2]–[4]. We are not aware of any reports describing such hematologic abnormalities in the mouse model of TB infection. The concentration of TAT in the BAL fluids from *M.tb-*infected mice was markedly lower in low TF mice as compared to the wild-type and HTF mice, indicating reduced activation of pulmonary coagulation in low TF mice. However, *M.tb* infection did not increase systemic coagulation in our mouse model as TAT concentration in the plasma of uninfected and *M.tb-*infected mice were similar in all genotypes. The exact reasons for the discrepancy between the mouse and human in developing thrombotic disorders in TB are unclear. Although the murine model of TB mimics the fundamental features of the human disease and share similarity in the innate and adaptive immune responses, it does not completely mirror the clinical TB disease in humans [52]–[54]. The differences between mouse and human in body size, lifespan, and species-specific physiological characteristics may result in differences in pathogenic features of TB infection between mice and humans. For example, despite the spectrum of lung pathology caused by TB infection is similar in mice and humans, the mouse lifespan may not provide enough time for the formation of true cavities [53]. It is possible that 2- to 8-weeks of infection may not be sufficient for necrosis of granuloma and release of TF expressing cells or TF microparticles from the granuloma into the circulation that could result in DIC and venous thrombosis in mice.

Our present data suggesting that TF may not play a significant role in host-defense during experimental TB is consistent with a recent report [55] where the role of blood coagulation in the host-defense in TB was investigated utilizing the mouse model. Kager et al. [55] found that overexpression or deficiency of endothelial cell protein C receptor (EPCR), a critical regulator of the protein C anticoagulant system, had no significant effect on *M.tb* growth, dissemination or inflammatory parameters. Additionally, overexpression of activated protein C (APC) or treatment with anti-APC antibodies had no significant impact on the inflammatory response induced by *M.tb* [55]. In an earlier study, the same group of investigators reported that mice with a mutation in thrombomodulin (TM) gene (TM^pro/pro^), which reduces APC generation, showed uncontrolled lung inflammation, elevated levels of pro-inflammatory cytokines, and higher bacterial dissemination to liver and spleen [56]. Despite strongly altered inflammatory response, TM^pro/pro^ mutation only modestly influenced the outgrowth of *M.tb* [56]. It may be pertinent to note here that TM^pro/pro^ mice not only have a severely impaired capacity to generate APC but also display an overall relative TM deficiency, including the function of the TM lectin domain [57] that is shown to exert host-protective anti-inflammatory effects [58]. Therefore, it is possible that not an altered coagulation but the impairment in the host-protective functions associated with the lectin domain may be responsible for an altered inflammatory response and bacterial dissemination in TM^pro/pro^ mice following *M.tb.* infection.

An interesting observation of the present study is that although the overall difference in TF levels in lung tissues of HTF and low TF mice, both uninfected and *M.tb-*infected, is 100-fold or more, the difference in TF levels in specific cell types or tissue regions may be much smaller between these mice. Immunohistochemical analysis of granulomas of wild-type, HTF and low TF mice revealed that TF expression in small, localized areas in granulomas of low TF mice was as high as observed in the granulomas of HTF mice. This data was further supported by *ex vivo* data where *M.tb.*-infected bone-marrow-derived macrophages of low TF mice exhibited ∼30% of TF activity relative to that observed in *M.tb*-infected bone marrow-derived macrophages of HTF mice. Interestingly, macrophages derived from different sources appear to respond differently to *M.tb* indicating that other factors within the macrophages influence differently how TF gene responds to various stimuli.

In conclusion, our present data suggest that TF does not play a significant role in TB pathogenesis. However, our data do not permit to rule out completely a role for TF in *M.tb* pathogenesis since *M.tb* induced a significant amount of TF expression in small localized areas in the granuloma even in low TF mice. It is possible that a small amount of TF expressed within the granuloma may be sufficient to mediate local coagulant and signaling functions to facilitate *M.tb.* growth and dissemination. Further studies with TF transgenic mice lacking TF completely in macrophages or other relevant specific cell types and/or mice lacking potential signaling partners to mediate TF signaling, and extended experimental time frame following the infection are needed to fully investigate the role of TF in TB pathogenesis. The present data also highlight the importance of exercising caution in interpreting the data obtained with low TF mice in experimental model systems since infectious agents or other pathophysiological agonists may induce a significant amount of TF expression in specific cell types or tissues in these mice.
